# Five-alpha reductase inhibitors and risk of prostate cancer among men with benign prostatic hyperplasia: A historical cohort study using primary care data

**DOI:** 10.12688/wellcomeopenres.19566.1

**Published:** 2023-07-11

**Authors:** Dixa B Thakrar, Ian J Douglas, Liam Smeeth, Krishnan Bhaskaran

**Affiliations:** 1 London School of Hygiene and Tropical Medicine, London, WC1E 7HT, UK

**Keywords:** Benign prostatic hyperplasia; prostate cancer; five-alpha reductase inhibitors; alpha blockers

## Abstract

**Background:** Five-alpha reductase inhibitors (5ARIs) are used in the management of benign prostatic hyperplasia (BPH). 5ARIs prevent the conversion of testosterone to dihydrotestosterone, which is important in prostate development. It has been suggested that 5ARIs can be used a chemopreventative agent for prostate cancer. The aim of this study was to assess the risk of prostate cancer associated with 5ARI use among men with BPH.

**Methods:** Using Clinical Practice Research Datalink (CPRD) from 1992 to 2011 in UK, prostate cancer risk was retrospectively compared in men with a new diagnosis of BPH, with no history of prostate cancer who were treated with 5ARIs, to men treated with alpha blockers (ABs) and those given no pharmacological treatment. Incidence rate of prostate cancer was calculated by treatment group; the association between BPH treatment group and prostate cancer was estimated by a multivariate Cox model.

**Results:** 77,494 men with newly diagnosed BPH were included. The crude incidence rate of prostate cancer was 892.4 cases per 100,000 person-years amongst those treated with 5ARIs, compared with 1209.0 and 1542.9 in those treated with ABs and untreated individuals, respectively. The HR adjusted for potential confounders was 0.79 (0.72-0.86) for 5ARI vs ABs and 0.72 (0.66-0.79) for 5ARI vs untreated. After excluding the first year after BPH diagnosis, adjusted HRs attenuated to 0.87 (0.79-0.97) for 5ARI vs ABs and 0.97 (0.87-1.08) for 5ARI vs untreated.

**Conclusion:** Among men diagnosed with BPH, we found evidence of lower risks of subsequent prostate cancer in those treated with 5ARIs, but this appeared to be driven by cases diagnosed within a year of BPH, possibly reflecting prevalent prostate cancers that were initially misdiagnosed. After excluding the first year after BPH diagnosis, there was little evidence of a reduced prostate cancer risk in those taking 5ARIs.

## Introduction

Prostate cancer is the commonest cancer in males in the UK, accounting for 26% of cancers diagnosed in men ^1^. Lifetime risk of a UK male developing prostate cancer is 1 in 6. Incidence of prostate cancer is 173.7 cases per 100,000 person-years in the UK. This has increased by 41% since the early 1990s and is projected to rise to 233 cases per 100,000 person-years by 2035 ^1^. Prostate cancer accounts for the second commonest cause of cancer related death among males; that is 13% of cancer deaths in males. Prostate cancer has a relatively long latency period ^2^. As such, preventative interventions could significantly improve the associated morbidity and mortality, as well as the costs of diagnosis and management.

Five-alpha reductase inhibitors (5ARIs) are used in the management of benign prostatic hyperplasia. 5ARIs prevent the conversion of testosterone to its active form, dihydrotestosterone, which is important in prostate development ^3^. It has been suggested that 5ARIs can be used as a chemopreventative agent for prostate cancer. Evidence from randomised trials suggests that treating healthy men with 5ARIs reduces the risk of prostate cancer. The Prostate Cancer Prevention Trial (PCPT) showed a 25% reduced relative risk of new prostate cancer among healthy men treated with finasteride for seven years compared to the placebo group ^4^. The REDUCE trial of 6729 men who were considered high risk for prostate cancer, due to age, elevated prostate-specific antigen (PSA), and a previous suspicion of prostate cancer, found a 23% relative risk reduction of developing prostate cancer among those treated with dutasteride for four years compared to placebo ^5^. A Cochrane review of eight studies, including the two aforementioned trials, evaluated the effect of 5ARIs on the prevention of prostate cancer. The review found a 25% relative risk reduction for prostate cancer in the treated compared to those not treated. Whilst these studies have shown a reduced risk of prostate cancer, this apparent benefit may have been driven by clinically insignificant disease that would not have been detected outside of a trial setting. In these studies, men were being regularly screened and hence the impact of 5ARI in men who are not routinely screened is unclear. Moreover, the trials did not necessarily study BPH patients specifically, who routinely are prescribed 5ARIs.

To date, the association of 5ARI use and prostate cancer risk has only sparsely been studied, with limited evidence available on whether the apparent benefits of 5ARIs are seen outside of a clinical trial, in the real-world setting. The aim of this study is to determine the risk of prostate cancer among men with BPH, comparing those treated with five-alpha reductase inhibitors to those treated using alpha-blockers (ABs) and those untreated, using data from a large database of routinely collected electronic primary care records. This is to determine if those on 5ARIs have a reduced risk of prostate cancer compared to those on alpha-blockers or conservative management.

## Methods

A historical cohort study was conducted of males with newly diagnosed BPH who were receiving 5ARI, alpha-blockers, or no treatment, between 1992-2010, using Clinical Practice Research Datalink (CPRD) GOLD.

### Data source

CPRD GOLD includes longitudinal electronic primary care health records for more than 14 million people from consenting GP practices in the UK since 1987 ^6^. CPRD is representative of the UK population in terms of age and sex. GPs provide anonymous data on demographics, physical findings, symptoms, diagnoses, referrals, hospital admissions, drug prescriptions, metrics, blood results, lifestyle practices and deaths, to the database ^7^. Data are collected prospectively for the purposes of routine clinical care; clinical findings and procedures including symptoms and diagnoses are recorded using a coded thesaurus of clinical terms known as Read codes ^8^. Numerous validation studies have shown the data to be of high quality across a range of outcomes ^9– 11^.

### Ethics and approvals

The study protocol was reviewed and approved by the Independent Scientific Advisory Committee (ISAC), an advisory body for the MHRA (approval number 12_044A, 19 ^th^ April 2013) and the London School of Hygiene and Tropical Medicine ethics committee (approval number 17130, 10 ^th^ June 2019). CPRD has overarching ethics approval for purely observational studies using anonymised data, which does not require individual patient consent.

### Study population

The study population comprised men with an incident diagnosis of BPH with no history of prostate cancer and were registered on CPRD between 1992 and 2010. Incident BPH was defined as a first READ code indicating BPH at least one year after start of follow-up in CPRD (an initial year of follow-up was required to avoid including prevalent cases coded within the first year of registration). Prior prostate cancer was also identified using relevant Read codes.

### Exposure and outcome

The exposure was defined as treatment for BPH: five-alpha reductase inhibitors; alpha-blocker or conservative management (neither drug), using GP prescription records. Exposure was determined initially at baseline, and time-updated during-follow-up, so that a patient could contribute person-time to more than one treatment group. Guidelines suggest that ABs are first line treatment, followed by 5ARIs ^12^, so our exposure definition allowed men to move from untreated to treated or from AB to 5ARI but not the reverse. Hence a patient treated with an AB who ceased treatment would remain in the AB group, and a patient ever treated with a 5ARI would remain in the 5ARI group regardless of subsequent changes. The outcome was prostate cancer, defined as a first Read code for prostate cancer during follow-up.

### Covariates

Additional data were collected, as part of CRPD, on age (categorised as <50, 50–54, 55–59, 60–64, 65–69, 70–74, 74–79, 80+ years), smoking (never, current, former smoker), alcohol (never, current, former drinker), body mass index (BMI, <18.5, 18.5-24.9, 25-29.9, 30+ kg/m2), ethnicity (White, Black, other), non-steroidal anti-inflammatory drugs (NSAIDS) and aspirin use, co-morbidities (previous cancer, diabetes, prostatitis), Index of Multiple Deprivation quintile (IMD, 1 = least deprived, 5 = most deprived) ^13^ and number of GP consultations in the year prior to BPH diagnosis (<5, 5–9, 10–19, 20–39, 40+).

### Analyses

Men with a new diagnosis of BPH were followed up from date of BPH diagnosis until date of diagnosis of prostate cancer, death, transfer out of CPRD or the end of the study period (end of 2011).

Continuous variables (age, year of BPH diagnosis, number of GP consultations) were grouped. Baseline characteristics of all variables were described by initial treatment group.

Crude prostate cancer incidence rates (IR) were calculated for each treatment group. A Cox model with an underlying timescale of time since BPH and time-dependent exposure status and age group was then fitted to calculate hazard ratios. A minimally adjusted model with only these covariates was first estimated. We then added all covariates listed above and with at least 80% complete data in the fully adjusted model. P-values were calculated using likelihood ratio tests. Collinearity was checked by looking at inflation of standard errors in the multivariate model.

Covariates with more than 20% missing data points (namely ethnicity) were investigated in a sensitivity analysis only to avoid significantly reducing the number of patients and statistical power in the main analysis.

A priori, it was decided to investigate effect modification of treatment by age (we collapsed the 5-year age bands into 10-year categories for the interaction, to reduce the number of parameters), smoking status, year of BPH diagnosis and deprivation. Interaction terms were fitted to the final multivariate Cox model and a likelihood ratio test was used to assess evidence for effect modification.

As solid cancers diagnosed within the first year of follow-up may represent prevalent cases that were initially misdiagnosed as BPH, the analysis was repeated excluding the first year of follow-up for all patients.

Analyses were conducted in Stata 16. Forest plots were generated in R using the Jasper package ^14^.

## Results

Between 1992 and 2010 there were 77,494 men with newly diagnosed BPH. Median age at diagnosis of BPH was 66.3 years (range 18.2–104.2). Table 1 presents the baseline characteristics. 54.2% were untreated at baseline, 34.2% were commenced on alpha-blockers and 11.6% on 5ARIs. Untreated men were younger than those on ABs, who were in turn younger than those on 5ARIs.

**Table 1.** Baseline characteristics of men with BPH by initial treatment group.

	Initial treatment group N (%)				
Baseline characteristics	Untreated	Alpha-blocker	5ARI	Total	p-value *
ALL	42,000	26,479	9,015	77,494	
**Age (years)**					<0.001
<50	3,235 (7.7)	1,207 (4.6)	214 (2.4)	4,656 (6.0)	
50-54	3,907 (9.3)	2,045 (7.7)	405 (4.5)	6,357 (8.2)	
55-59	6,171 (14.7)	3,501 (13.2)	926 (10.3)	10,598 (13.7)	
60-64	7,511 (17.9)	4,756 (18.0)	1,452 (16.1)	13,719 (17.7)	
65-69	7,348 (17.5)	4,687 (17.7)	1,608 (17.8)	13,643 (17.6)	
70-74	6,116 (14.6)	4,349 (16.4)	1,661 (18.4)	12,126 (15.7)	
75-79	4,341 (10.3)	3,184 (12.0)	1,379 (15.3)	8,904 (11.5)	
80+	3,371 (8.0)	2,750 (10.4)	1,370 (15.2)	7,491 (9.7)	
Median (IQR)	66.1 (58.9-73.6)	67.8 (60.9-75.2)	70.7 (63.5-77.6)	67.3 (60.1-74.7)	
**Year of BPH diagnosis**					<0.001
1992-1999	15,377 (36.6)	5,623 (21.2)	2,393 (26.5)	23,393 (30.2)	
2000-2004	13,469 (32.1)	9,445 (35.7)	2,392 (26.5)	25,306 (32.7)	
2005-2010	13,154 (31.3)	11,411 (43.1)	4,230 (46.9)	28,795 (37.2)	
**Smoking status**					<0.001
Never	20,683 (49.3)	11,743 (44.4)	4,145 (46.0)	36,571 (47.2)	
Current	10,501 (25.0)	6,521 (24.6)	1,880 (20.9)	18,902 (24.4)	
Former	9,990 (23.8)	7,907 (29.9)	2,854 (31.7)	20,751 (26.8)	
Missing	826 (2.0)	308 (1.2)	136 (1.5)	1,270 (1.6)	
**Alcohol status**					<0.001
Never	4,536 (10.8)	3,199 (12.1)	1,190 (13.2)	8,925 (11.5)	
Current	33,451 (79.7)	20,840 (78.7)	6,956 (77.2)	61,247 (79.0)	
Former	607 (1.5)	640 (2.4)	224 (2.5)	1,471 (1.9)	
Missing	3,406 (8.1)	1,800 (6.8)	645 (7.2)	5,851 (7.6)	
**Body mass index (kg/m ^2^)**					<0.001
<18.5	308 (0.7)	195 (0.7)	79 (0.9)	582 (0.8)	
18.5-24.9	13,550 (32.3)	8,170 (30.9)	2,890 (32.1)	24,610 (31.8)	
25-29.9	18,042 (43.0)	11,470 (43.3)	3,871 (42.9)	33,383 (43.1)	
30+	6,377 (15.2)	4,793 (18.1)	1,533 (17.0)	12,703 (16.4)	
Missing	3,723 (8.9)	1,851 (7.0)	642 (7.1)	6,216 (8.0)	
**Previous Prostatitis**					0.526
No	40,773 (97.1)	25,739 (97.2)	8,746 (97.0)	75,258 (97.1)	
Yes	1,227 (2.9)	740 (2.8)	269 (3.0)	2,236 (2.9)	
**Ethnic group**					0.986
White	7,492 (17.8)	5,559 (21.0)	1,960 (21.7)	15,011 (19.4)	
Black	137 (0.3)	97 (0.4)	33 (0.4)	267 (0.3)	
Other	3,525 (8.4)	2,586 (9.8)	917 (10.2)	7,028 (9.1)	
Missing	30,846 (73.4)	18,237 (68.9)	6,105 (67.7)	55,188 (71.2)	
**Aspirin use**					<0.001
No	33,460 (79.7)	19,106 (72.2)	6,118 (67.9)	58,684 (75.7)	
Yes	8,540 (20.3)	7,373 (27.8)	2,897 (32.1)	18,810 (24.3)	
**NSAID use**					<0.001
No	19,034 (45.3)	10,026 (37.9)	3,524 (39.1)	32,584 (42.1)	
Yes	22,966 (54.7)	16,453 (62.1)	5,491 (60.9)	44,910 (58.0)	
**History of other cancers**					<0.001
No	38,904 (92.6)	24,289 (91.7)	8,070 (89.5)	71,263 (92.0)	
Yes	3,096 (7.4)	2,190 (8.3)	945 (10.5)	6,231 (8.0)	
**Diabetes**					<0.001
No	38,828 (92.5)	23,910 (90.3)	8,046 (89.3)	70,784 (91.3)	
Yes	3,172 (7.6)	2,569 (9.7)	969 (10.8)	6,710 (8.7)	
**Number GP consultations in year before BPH diagnosis**					<0.001
<5	11,143 (26.5)	3,753 (14.2)	836 (9.3)	15,732 (20.3)	
5-9	9,667 (23.0)	5,863 (22.1)	1,645 (18.3)	17,175 (22.2)	
10-19	12,891 (30.7)	9,036 (34.1)	3,222 (35.7)	25,149 (32.5)	
20-39	7,179 (17.1)	6,551 (24.7)	2,714 (30.1)	16,444 (21.2)	
40+	1,120 (2.7)	1,276 (4.8)	598 (6.6)	2,994 (3.9)	
**IMD score**					<0.001
1 (most deprived)	9,791 (23.3)	5,843 (22.1)	1,932 (21.4)	17,566 (22.7)	
2	9,285 (22.1)	5,596 (21.1)	1,892 (21.0)	16,773 (21.6)	
3	7,592 (18.1)	4,651 (17.6)	1,590 (17.6)	13,833 (17.9)	
4	11,020 (26.2)	7,017 (26.5)	2,419 (26.8)	20,456 (26.4)	
5 (least deprived)	4,312 (10.3)	3,372 (12.7)	1,182 (13.1)	8,866 (11.4)	

*p-value: chi-squared test; 5ARI, five-alpha reductase inhibitor; BPH, benign prostatic hyperplasia; NSAID, non-steroidal anti-inflammatory, GP, general practitioner; IMD, index of multiple deprivation

There was a large amount of missing data for ethnicity (71% missing). Those with missing ethnicity, BMI, smoking or alcohol status were on average two years older at BPH diagnosis than those without missing values. Those with missing data were also diagnosed earlier in calendar time.

During the study, 39.9% remained untreated throughout, compared to more than 50% at baseline. 21.5% received a 5ARI at some point during follow-up, with or without previous AB, compared to 11.6% at baseline ( Table 2).

**Table 2.** Treatment history of BPH throughout the study period.

Treatment combination	N	%
**Untreated**	30931	39.9
**Alpha-blocker only**	29899	38.6
**5ARI only**	10948	14.1
**AB + 5ARI**	5716	7.4
**Total receiving 5ARI**	16664	21.5

BPH, benign prostatic hyperplasia; AB, alpha-blocker; 5ARI, five-alpha reductase inhibitor

There were 6537 (8.4%) cases of prostate cancer ( Table 3). Of these cases, 3273 (50.1%) occurred within one year of BPH diagnosis. Total follow-up time was 495,716 person-years. Average follow-up was 6.4 years. Crude incidence rate of prostate cancer was 1318.7 cases per 100,000 person-years (95% CI 1287.1-1351.1). Incidence among those on 5ARIs with no history of AB were almost identical to those treated with both (892.5 and 892.1 cases per 100,000 person-years respectively) so these patients were combined in the rest of the analysis (overall incidence: 892.4, 829.4-960.1). By comparison, incidences were 1209.0 per 100,000 person years (1158.7-1261.5) among those on alpha-blockers and 1542.8 (1493.8-1593.4) among untreated patients.

**Table 3.** Univariate analysis showing incidence and incident rate ratios of prostate cancer by exposure variable among men with BPH.

Exposure	PC Cases	Incidence rate (per 100,000 person years)	95%CI	IRR	95%CI	p-value
ALL	6537	1318.7	1287.1-1351.1			
**Treatment**						<0.001
Untreated	3691	1542.8	1493.8-1593.4	1.00	(ref)	
AB only	2128	1209.0	1158.7-1261.5	0.78	0.74-0.83	
5ARI only	539	892.5	820.2-971.1	0.58	0.53-0.63	
5ARI + AB	179	892.1	770.5-1032.9	0.58	0.50-0.67	
5ARI ± AB	718	892.4	829.4-960.1	0.64	0.59-0.69	
**Age (years)**						<0.001
<50	24	158.3	106.1-236.2	1.00	(ref)	
50–54	103	423.8	349.4-514.2	2.68	1.72-4.17	
55–59	389	811.8	735.0-896.6	5.12	3.39-7.74	
60–64	803	1086.0	1013.5-1163.8	6.86	4.57-1029	
65–69	1172	1345.5	1270.6-1424.8	8.50	5.67-12.73	
70–74	1420	1595.0	1514.2-1680.2	10.07	6.73-15.08	
75–79	1321	1734.2	1643.2-1830.3	10.95	7.31-16.40	
80+	1305	1589.6	1505.7-1678.2	10.04	6.70-15.03	
**Year of BPH diagnosis**						<0.001
1992–1999	2586	1165.4	1121.3-1211.2	1.00	(ref)	
2000–2004	2122	1208.6	1158.2-1261.1	1.04	0.98-1.10	
2005–2010	1829	1861.9	1778.5-1949.2	1.60	1.50-1.70	
**Smoking status**						0.002
Never	3235	1304	1259.9-1349.7	1.00	(ref)	
Current	1568	1223.1	1164.0-1285.1	0.94	0.88-1.00	
Former	1594	1390.8	1324.2-1460.8	1.07	1.00-1.13	
**Alcohol status**						0.571
Never	767	1337.8	1246.4-1435.9	1.00	(ref)	
Current	5145	1284.1	1249.5-1319.7	0.96	0.89-1.04	
Former	94	1306.4	1067.3-1599.1	0.98	0.79-1.21	
**Body mass index (kg/m ^2^)**						<0.001
<18.5	40	1214.31	890.72-1655.5	0.96	0.70-1.32	
18.5-24.9	2071	1261.68	1208.5-1317.2	1.00	(ref)	
25-29.9	2963	1344.79	1297.2-1394.1	1.07	1.01-1.13	
30+	869	1137.68	1064.5-1215.9	0.90	0.83-0.98	
**Previous prostatitis**						<0.001
No	6388	1329.19	1297.0-1362.2	1.00	(ref)	
Yes	149	985.23	839.08-1156.8	0.74	0.63-0.87	
**Ethnicity**						<0.001
White	1135	1106.1	1043.6-1172.3	1.00	(ref)	
Black	34	2350.6	1679.6-3289.7	2.13	1.51-2.99	
Other	532	1106.8	1016.6-1204.9	1.00	0.90-1.11	
**Aspirin use**						<0.001
No	5004	1257.4	1223.0-1292.7	1.00	(ref)	
Yes	1533	1568.3	1491.7-1648.8	1.25	1.18-1.32	
**NSAID use**						0.138
No	2873	1291.9	1245.5-1340.0	1.00	(ref)	
Yes	3664	1340.5	1297.8-1384.6	1.04	0.99-1.09	
**Previous cancer**						<0.001
No	5915	1280.6	1248.4-1313.6	1.00	(ref)	
Yes	622	1839.1	1700.1-1989.5	1.44	1.32-1.56	
**Number of GP consultations in previous year**						<0.001
<5	1334	1132.9	1073.7-1195.3	1.00	(ref)	
5–9	1481	1244.2	1182.5-1309.2	1.10	1.02-1.18	
10–19	2240	1411.2	1354.0-1470.9	1.25	1.16-1.33	
20–39	1315	1499.5	1420.6-1582.7	1.32	1.23-1.43	
40+	167	1335.2	1147.3-1553.9	1.18	1.00-1.38	
**Diabetes**						0.934
No	6061	1318.3	1285.5-1351.9	1.00	(ref)	
Yes	476	1323.5	1209.8-1447.9	1.00	0.91-1.10	
**IMD score**						0.208
1	1570	1362.7	1296.9-1431.8	1.00	(ref)	
2	1393	1271.9	1206.8-1340.5	0.93	0.87-1.00	
3	1193	1349.4	1275.0-1428.2	0.99	0.92-1.07	
4	1660	1325.9	1263.6-1391.2	0.97	0.91-1.04	
5	721	1256.7	1168.2-1351.9	0.92	0.84-1.01	

PC, prostate cancer; AB, alpha-blocker; 5ARI, five-alpha reductase inhibitor; 95%CI, 95% confidence interval; IRR, incidence rate ratio; BPH, benign prostatic hyperplasia; NSAID, non-steroidal anti-inflammatory, GP, general practitioner; IMD, index of multiple deprivation. Age refers to current (time-updated) age.

Univariate analysis showing IR and crude IRRs of prostate cancer by all covariates are displayed in Table 3. The unadjusted (i.e. controlling for the underlying timescale only), age-adjusted, and fully adjusted associations between 5ARI use and prostate cancer from Cox models are shown in Table 4, and estimates for all covariates from the fully adjusted model are in Table 5. The risk of prostate cancer was lower in the 5ARI group compared with both AB users (adjusted HR 0.79, 0.72-0.86) and untreated patients (adjusted HR 0.72, 0.66-0.79). Adding ethnicity in a sensitivity analysis among those with non-missing data reduced the number of individuals in the analysis to 21,088 but the pattern of results was consistent with the main analysis (HR 0.70, 0.60-0.83 for 5ARI vs AB and 0.68, 0.58-0.80 for 5ARI vs untreated).

**Table 4.** Multivariate time dependent Cox model showing the hazards ratio for prostate cancer among men with BPH.

Model specification	HR (95% CI)	
	5ARI vs AB	5ARI vs untreated
**Unadjusted**	0.85 (0.78-0.92)	0.83 (0.77-0.90)
**Adjusted for current age only**	0.78 (0.71-0.85)	0.71 (0.65-0.77)
Adjusted for all covariates *	0.79 (0.72-0.86)	0.72 (0.66-0.79)

HR, hazard ratio; AB, alpha-blocker; 5ARI, five-alpha reductase inhibitor; * adjusted for age, smoking, alcohol, body mass index, non-steroidal anti-inflammatory drugs and aspirin use, co-morbidities (previous cancer, diabetes, prostatitis), Index of Multiple Deprivation quintile, and number of GP consultations in the year prior to BPH diagnosis

**Table 5.** Fully adjusted Cox model for association between 5ARI use and prostate cancer showing hazard ratios for all covariates.

Covariate	HR	95%CI	p-value
**Treatment**			
5ARI vs AB only	0.79	0.72-0.86	
5ARI vs untreated	0.72	0.66-0.79	
**Current age (years)**			<0.001
<50	1.00	(ref)	
50-54	2.43	1.52-3.90	
55-59	5.74	3.73-8.83	
60-64	8.59	5.62-13.14	
65-69	11.95	7.83-18.25	
70-74	15.72	10.30-24.00	
75-79	18.33	12.00-28.00	
80+	18.90	12.36-28.90	
**Year of BPH diagnosis**			<0.001
1992-1999	1.00	(ref)	
2000-2004	1.04	0.98-1.12	
2005-2010	1.24	1.15-1.33	
**Smoking status**			0.035
Never	1.00	(ref)	
Current	1.00	0.94-1.07	
Former	0.92	0.86-0.98	
**Alcohol status**			0.808
Never	1.00	(ref)	
Current	1.02	0.94-1.10	
Former	0.96	0.77-1.19	
**BMI (kg/m ^2^)**			<0.001
<18.5	0.77	0.55-1.07	
18.5-24.9	1.00	(ref)	
25-29.9	1.12	1.05-1.18	
30+	1.00	0.92-1.09	
**Previous prostatitis**	0.94	0.79-1.11	0.471
**Aspirin use**	0.91	0.85-0.97	0.006
**NSAID use**	1.03	0.97-1.08	0.369
**History of other cancer**	1.08	0.98-1.18	0.109
**Diabetes**	0.87	0.78-0.96	0.005
**Number of GP consultations in previous year**			<0.001
<5	1.00	(ref)	
5-9	0.95	0.87-1.03	
10-19	0.95	0.88-1.02	
20-39	0.88	0.80-0.96	
40+	0.70	0.59-0.84	
**IMD score**			0.156
1	1.00	(ref)	
2	0.91	0.84-0.98	
3	0.96	0.88-1.04	
4	0.94	0.87-1.01	
5	0.95	0.86-1.04	

HR, hazard ratio; AB, alpha-blocker; 5ARI, five-alpha reductase inhibitor; 95%CI, 95% confidence interval; NSAID, non-steroidal anti-inflammatory, GP, general practitioner; IMD, index of multiple deprivation

**Figure 1.**
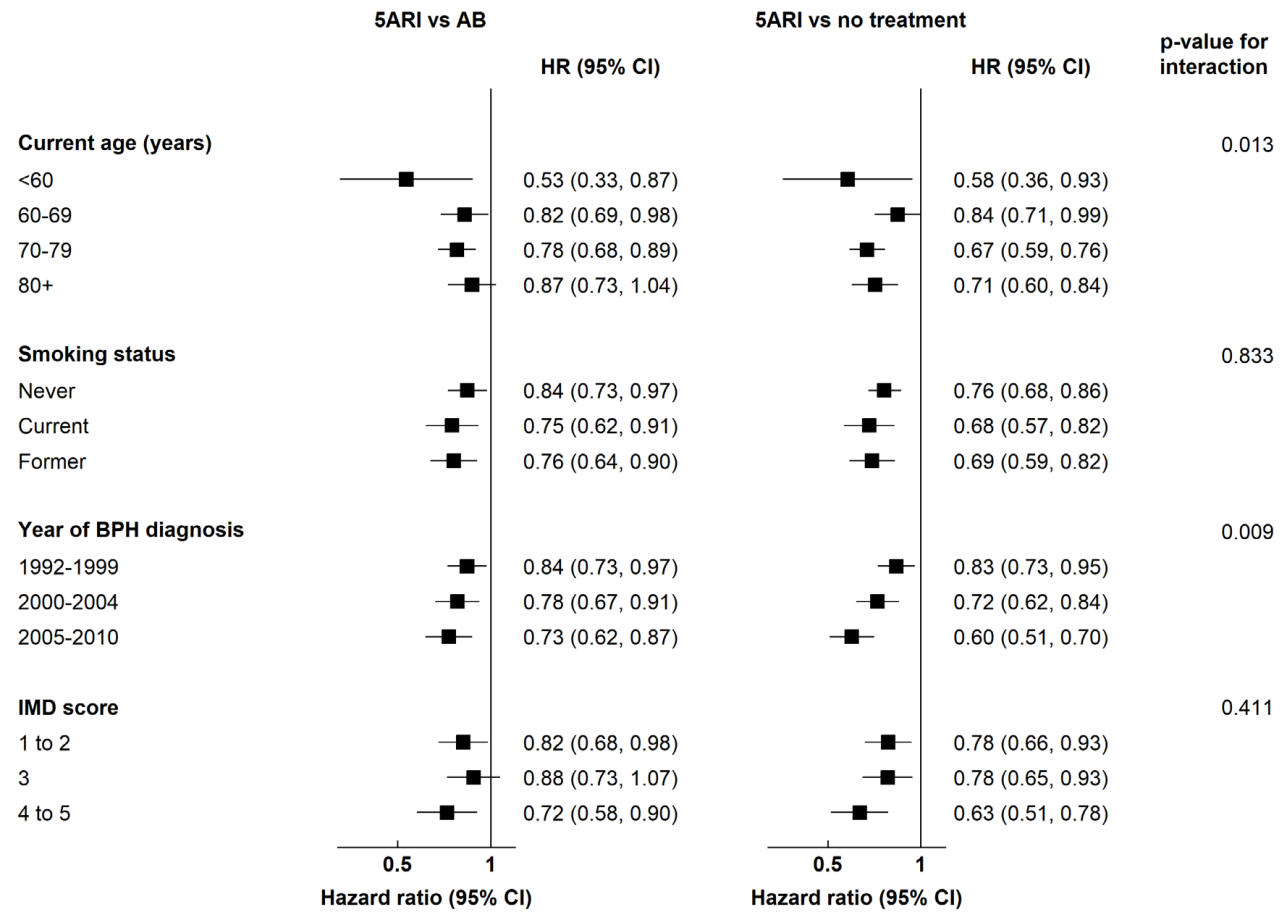
Adjusted HRs for association between treatment group and prostate cancer, stratified by other factors. HR, hazard ratio; 5ARI, five-alpha reductase inhibitor; 95% CI, 95% confidence interval; BPH, benign prostatic hyperplasia; IMD, index of multiple deprivation

A priori it was decided to investigate the possible effect modification of treatment by age, smoking status, year of BPH diagnosis and deprivation ( Figure 1). As there were no cases of prostate cancer amongst those less than 50 years on 5ARI, this group was combined with those aged 50-59 years in the interaction term. There was some evidence of effect modification by age (p=0.013) but no clear trend in the estimates. There was also some evidence that the inverse association between 5ARI use and prostate cancer risk was greater amongst those who were diagnosed with BPH in later years (p=0.009). There was no evidence of effect modification by smoking or deprivation.

Approximately half of the cases occurred early on during the follow up period, which might represent prevalent prostate cancer cases that were misdiagnosed as BPH. The analysis was repeated, excluding the first year of follow-up and hence excluding prostate cancer outcomes recorded in that period. Follow-up time now began at 1-year after BPH diagnosis. There were 2952 (45.2%) prostate cancer cases remaining in this analysis among a cohort of 65,862 (85.0% of the original cohort). The associations between treatment group and prostate cancer risk substantially attenuated in this analysis (HR 0.87, 0.79-0.97 for 5ARI vs AB and 0.97, 0.87-1.08 for 5ARI vs untreated).

## Discussion

With 77,494 subjects and 6537 prostate cancer cases we conducted one of largest studies to date investigating the association between 5ARI use and prostate cancer risk. The initial analysis revealed that BPH patients on 5ARIs had 28% lower risk of developing prostate cancer than those who did not take any BPH treatment, and 21% lower risk compared to those taking alpha-blockers, after adjusting for potential confounders. However, an unexpected observation was that half of the prostate cancer cases occurred within one year of BPH diagnosis. After excluding these patients, who may have had a prevalent cancer misdiagnosed as BPH, there was no evidence that 5ARIs were associated with a reduced risk of prostate cancer compared with no treatment. A slightly lower risk in 5ARI users compared to alpha-blocker users seemed to reflect the latter group having somewhat higher risk than the untreated group, rather than any protective effect of 5ARIs.

Overall, this cohort had a higher incidence rate of prostate cancer compared to the national average: 1318.7 vs 173.7 cases per 100,000 person-years ^1^. After excluding cases diagnosed within the first year, incidence fell to 660.1. A plausible explanation is that those with BPH are likely to have more investigations for prostate cancer, such as regular PSA testing or digital rectal examinations, which would increase detection rates. At present, BPH is not considered a risk factor for prostate cancer ^15^.

Clinical trials and a systematic review have shown a 23-25% relative risk reduction in prostate cancer among those who took 5ARIs compared to those who did not ^4, 5, 16^. Whilst these results are similar to the initial analysis, the trial participants were healthy and not BPH patients, making comparison difficult ^4, 5^. The REDUCE and PCPT trials however, found that 5ARIs were associated with higher incidence of high-grade cancers. Furthermore, trials are often limited to shorter follow-up, unlike observational studies. To our knowledge, this is the first study using large, routinely collected, real world data in the UK to examine the effect of BPH treatment on the risk of prostate cancer in men with BPH. The advantage of observational data is that it allows for studying the effect in a real-world setting rather than within the stringent inclusion and exclusion criteria of a clinical trial.

A 20-year retrospective cohort study of 24,012 men with BPH, in Canada, from 1995–2014, found that 5ARI users had a 40% reduced prostate cancer risk compared to non-users ^17^. A prospective cohort study of 38,058 male healthcare professionals in the United States found HR, for prostate cancer, associated with exposure to 5ARI compared to untreated men was 0.77 (95%CI 0.65-0.91) ^18^. However, as this study was limited to healthcare professionals, it may not be generalisable to the general population. A Swedish population-based case-control study using national registries, compared over 26,000 cases of prostate cancer with over 130,000 controls. Odds ratio, for prostate cancer, comparing 5ARI to no treatment was 0.89 (95%CI 0.84-0.94) ^19^. These studies are largely consistent with our initial analysis ( Table 6). None of these studies excluded patients diagnosed with prostate cancer within one year of BPH diagnosis. 

**Table 6.** Comparison of studies investigating effect of 5ARIs on prostate cancer risk.

Study	Location	Design	Relative risk estimate (5ARI vs. non-users)	95%CI
**PCPT, 2003 ^4^**	USA	RCT	0.77	0.70-0.85
**REDUCE, 2010 ^5^**	USA	RCT	0.75	0.69-0.81
**Van Rompay, 2019 ^17^**	Canada	Cohort	0.60	0.52-0.68
**Preston, 2014 ^18^**	USA	Cohort	0.77	0.65-0.91
**Robinson, 2013 ^19^**	Sweden	Case-control	0.89	0.84-0.94
**Murtola, 2007 ^20^**	Finland	Case-control	1.41	1.31-1.51
***Present study***	***UK***	***Cohort***	***0.79 (vs AB)*** ***0.72 (vs untreated)***	***0.72-0.86*** ***0.66-0.79***
***Present study*** ***(removing first year*** ***of follow-up)***	***UK***	***Cohort***	***0.87 (vs AB)*** ***0.97 (vs untreated)***	***0.79-0.97*** ***0.87-1.08***

AB, alpha-blockers; 5ARI, five-alpha reductase inhibitors; 95%CI, 95% confidence intervals; PCPT, prostate cancer prevention trial; RCT, randomised control trial.

A case-control study in Finland, using Finnish national registries found that overall prostate cancer risk was increased amongst those who used either alpha-blockers or 5ARIs compared with non-users (AB odds ratio: 1.79, 95%CI: 1.67-1.91; 5ARI odds ratio: 1.41; 95%CI 1.31-1.51) ^20^. However, they additionally found that risk was lower amongst 5ARI users compared to AB users: OR 0.80 (95%CI 0.64-1.00). The authors suggested that this apparent increased risk may be due to enhanced diagnostics of prostate cancer in BPH patients. This may be true in our cohort where the overall incidence of prostate cancer was higher than the national average, and that those on alpha-blockers had an increased prostate cancer risk compared to those not treated. By definition, those commenced on treatment would have symptomatic BPH and hence confounding by indication may have affected the results.

Whilst it is unclear whether BPH increases prostate cancer risk, the symptoms associated with BPH may lead to increased surveillance, investigation and hence increased prostate cancer detection amongst these patients. As patients typically start on alpha-blockers, prostate cancer cases, particularly residual prevalent cases even after excluding first the year, are more likely to be picked up during the period when they are on alpha-blockers. By the time they have been started on 5ARIs, they may have already undergone the necessary investigation for prostate cancer, by which time it has been ruled out. This may explain the observed higher prostate cancer risk in AB users, compared to those not treated or on 5ARIs.

The study’s main strength is the use of CPRD, a large, validated longitudinal primary care cohort. It is known for having a high accuracy of diagnoses and completeness of prescriptions, which provides assurance that BPH, interventions and outcomes would have been coded appropriately ^9, 21^. Data in CPRD is collected without study hypothesis which reduces risk of recall bias. Several potential confounders were controlled for in the analyses. With a cohort of over 70,000, we were able to examine the possible effect modification in the association between treatment and prostate cancer, without losing power and have almost 20-years of follow-up. This is not always possible in a clinical trial. We controlled for year of BPH diagnosis to control for potential changes in treatment guidelines and availability and prescribing patterns, which may have changed with time.

The study has limitations. Firstly, we did not carry out validation of the cases. It has been shown that prostate cancer may be under-documented in CPRD by 18.6% compared with cancer registry data ^22^. It is not clear how this misclassification of outcome may impact the results, as exclusion may be random, or nondifferential, in which case, there would be little effect on the result. The study can be enhanced by validating a sample of cases with cancer registry data.

Secondly, data from CPRD on prescriptions indicate whether the prescription has been issued and not whether the medication has been dispensed by a pharmacy or taken by the patient. Hence, there may have also been misclassification, possibly non-differential, of exposure status. Therefore, we may have over or underestimated the effect of BPH treatment in reducing prostate cancer risk. Furthermore, patients went from untreated to alpha-blocker treatment to 5ARI treatment and could not go from 5ARI to alpha-blocker treatment. Whilst alpha-blockers are first-line, and hence unlikely to be commenced after 5ARI, it is possible that this may have happened in some patients and was not accurately reflected in the data procurement from the CPRD database. Patients may also have been on treatments simultaneously. Patients could additionally have stopped treatment, possibly due to the side effects, and therefore become untreated again, which would not have been accurately reflected in the data.

CPRD does not collect information on grade and hence we could not examine the effect of treatment on the grade of prostate cancers diagnosed in patients on 5ARIs, as noted previously ^4, 5^. Moreover, PSA data and information on family history of prostate cancer were not available, which may have been useful to control for.

Black ethnicity is a known risk factor for prostate cancer ^23^. More than 70% of the patients had missing ethnicity, and hence ethnicity was not included in the final model to ensure statistical power could be maintained. However, our sensitivity analysis including ethnicity among those with data suggested that missingness did not materially impact the effect of treatment and hence the interpretation of the results. Missingness of other variables, namely alcohol, smoking and BMI, was relatively small (≤8%) and, and in cohort of this size, did not materially impact the main results.

## Conclusions and recommendations

Evidence of lower risks of prostate cancer in men with BPH treated with 5ARIs compared with ABs/no treatment appeared to be driven by cases diagnosed within a year of BPH, which likely include prevalent prostate cancers that were initially misdiagnosed. After excluding the first year after BPH diagnosis, there was little evidence of a reduced prostate cancer risk in those taking 5ARIs, suggesting that 5ARIs may have limited potential to protect against cancer in this clinical setting. Future studies should investigate the effect of treatment on mortality from prostate cancer and grade of prostate cancer, to better inform clinical decision making.

## Data Availability

The patient data in this study are provided by the Clinical Practice Research Datalink (CPRD) obtained under licence from the UK Medicines and Healthcare products Regulatory agency (MHRA). This data is only available upon approval of an application to the CPRD. Information about how to apply for data can be found at https://cprd.com/data-access. Applicants must register on the submission system, supply curriculum vitae information, and complete a research protocol, which is then reviewed by an independent committee. The specific dataset we accessed was CPRD GOLD, as outlined in the Methods section. figshare: STROBE checklist for ‘Five-alpha reductase inhibitors and risk of prostate cancer among men with benign prostatic hyperplasia: A historical cohort study using primary care data’. https://doi.org/10.6084/m9.figshare.23255204.v1 ^24^
