# Supplementary material for: Five-alpha reductase inhibitors and risk of prostate cancer among men with benign prostatic hyperplasia: A historical cohort study using primary care data
Source: Wellcome Open Res. 2023 Jul 11;8:295. [Version 1] doi: 10.12688/wellcomeopenres.19566.1 (PMC11106599; doi:10.12688/wellcomeopenres.19566.1)
Supplement: Peer review history

# Reviewer report: Reviewer response for version 1

**DOI:** 10.21956/wellcomeopenres.21676.r79900

**Published:** 2024-05-20

**Authors:** Blánaid Hicks

**Affiliations:** 1 Queen's University Belfast, Belfast, Northern Ireland, UK

This study involves a large cohort of men diagnosed with benign prostatic hyperplasia in the CPRD (1992-2010) with follow up until 2011. Analyses compared five alpha reductase inhibitor use to alpha-blocker and non-treatment and the risk of prostate cancer, addressing an important question. In general, the study was well conducted and clearly described. The authors have placed their findings in context. However, I do have a number of queries and suggestions that could be considered.

 1.  The authors should consider using a lag period for their exposure definition, at least in sensitivity analyses. Under the current exposure definition, the authors appropriately used a time varying approach, allowing patients to move through exposure groups. However, if a cancer event occurred after a short exposure period to 5ARI (or AB) this would be considered an exposed event, and seems biologically implausible given what we know about cancer latency. For example, if a hypothetical patient took an alpha-blocker for 4 years, subsequently switched to a 5ARI and was diagnosed with cancer within 6 months of switch this would be considered a 5ARI event. To appropriately deal with this, exposure should be lagged for e.g. by one year whereby a patient is considered exposed 1 year after their switch.  

 2. The data available from CPRD would allow for dose response relation to be investigated e.g. DDDs and or models evaluating number of prescriptions, time since initiation etc. Such dose response analyses, including for example flexible models can be very useful to evaluate the HRs over time, to identify any dose response and in particular in this setting, can be useful for identifying and/or hypothesising about potential biases that may be affecting results.

 3. The presentation of results in the tables could be improved. It is not clear why Table 3 and Table 5 present IRRs/HRs for all confounders, as this was not a stated aim of the study and indeed it is not recommended (Table 5), due to Table 2 Fallacy. Table 3 may be useful as an appendix. For the main analyses, the number of events, person years etc. should be included, so that the readership has full information on the analyses conducted.

 4.  AB and 5ARIs are also indicated for conditions other than BPH (hypertension, male pattern baldness etc). Did the authors consider this in their analyses e.g. remove those with a history of medication use prior to first diagnosis of BPH?

 5. A minor clarification would help regarding “an initial year of follow-up was required”. Does this refer at least 1-year history prior to BPH diagnosis (i.e. 1991 for those diagnosed in 1992) or 1-year follow-up post BPH (in which case no cohort member could therefore die during this period)?

 6. I agree with the assessment that as the treatment of BPH varies by severity and symptoms, plus the difference in action of these drugs, that these treatment groups (and non-treated group) are very different and make comparisons difficult. Unfortunately, factors such as PSA, volume etc. are not available. While discussed in  their paper and the authors have adjusted for a number of confounders and investigate effect modification, could they consider for example some methods such as high dimensional propensity scores as a sensitivity analyses?

Is the work clearly and accurately presented and does it cite the current literature?

Yes

If applicable, is the statistical analysis and its interpretation appropriate?

Yes

Are all the source data underlying the results available to ensure full reproducibility?

Partly

Is the study design appropriate and is the work technically sound?

Yes

Are the conclusions drawn adequately supported by the results?

Yes

Are sufficient details of methods and analysis provided to allow replication by others?

Yes

Reviewer Expertise:

pharmacoepidemiology\cancer epidemiology

I confirm that I have read this submission and believe that I have an appropriate level of expertise to confirm that it is of an acceptable scientific standard, however I have significant reservations, as outlined above.

# Reviewer report: Reviewer response for version 1

**DOI:** 10.21956/wellcomeopenres.21676.r74821

**Published:** 2024-04-29

**Authors:** Chris R. Cardwell

**Affiliations:** 1 Queen's University Belfast, Belfast, UK

The manuscript describes a large cohort study conducted within the Clinical Practice Research Datalink comparing prostate cancer risk in men using 5ARIs to men using alpha blockers and those untreated. The study addresses an important clinical question. The study is well conducted. The analysis is appropriate, clear and well described. The interpretation is appropriate and is placed in context.

 Minor questions and comments are:

 1. I would have expected a lag to be included in the exposure definition because it does not seem particularly plausible that 5ARIs would instantaneously increase prostate cancer risk after initiating treatment. I appreciate this would be addressed to some extent in the analysis excluding the first year after prostate cancer diagnosis. Regardless, a discussion of the benefits or otherwise of using a lag would be useful.

 2. It is not clear what happens to individuals who used 5ARIs before their BPH diagnosis.

 3. It appears that when all covariates are added to the model, the analysis is conducted on a complete case basis. If so, this should be more clearly stated.

 4. Historically, based upon the trials, there was concern that 5ARIs could cause high-risk prostate cancer (Theoret MR, et. al., 2011 [Ref 1]). Unfortunately, this cannot be investigated in this dataset. The inability to identify high-risk disease is noted as a weakness but I think more context should be given to this.

 5.In Table 3, the “5ARI only” group HR is 0.58 and the “5ARI + AB” the HR is 0.58. However, in the “5ARI ± AB” group the HR is 0.64 (and not 0.58 as might be expected). I think this could be correct but, I think, is worth checking.

 6. The identification of BPH using GP diagnosis codes is a key part of the study. For instance, it seems more likely that a patient with both a BPH diagnosis code and a 5ARI prescription has BPH than a patient with only a single BPH diagnosis code. Are there any studies that have investigated how accurate GP diagnosis codes are at identifying BPH?

 7. The analysis excluding the first year after BPH diagnosis informs the conclusion but is only mentioned in the final few lines of the results. Arguably more space in the results could be devoted to this analysis. For instance, could a table be included showing numbers of events and person years etc.

Is the work clearly and accurately presented and does it cite the current literature?

Yes

If applicable, is the statistical analysis and its interpretation appropriate?

Yes

Are all the source data underlying the results available to ensure full reproducibility?

No source data required

Is the study design appropriate and is the work technically sound?

Yes

Are the conclusions drawn adequately supported by the results?

Yes

Are sufficient details of methods and analysis provided to allow replication by others?

Yes

Reviewer Expertise:

Statistics \ Epidemiology.

I confirm that I have read this submission and believe that I have an appropriate level of expertise to confirm that it is of an acceptable scientific standard.

## References

[1] : The risks and benefits of 5α-reductase inhibitors for prostate-cancer prevention. *N Engl J Med* .2011;365(2) : 10.1056/NEJMp1106783 97-9 10.1056/NEJMp1106783 21675880

# Reviewer report: Reviewer response for version 1

**DOI:** 10.21956/wellcomeopenres.21676.r72933

**Published:** 2024-02-26

**Authors:** Mette Nørgaard

**Affiliations:** 1 Aarhus university, Aarhus, Denmark

The current study examines Five-alpha reductase inhibitors (5-ARI) and risk of prostate cancer among men with benign prostatic hyperplasia using alpha-blockers and no BPH related treatment, respectively as comparisons.

 The study is a historical cohort study using primary care data. It is a rather old study – follow-up ended in 2011.  Why did the authors find it relevant to report on more than 12 year old data? Much has changed regarding both BPH and prostate cancer diagnostics over the years.

 Potential chemoprevention of prostate cancer by 5-ARI treatment has been discussed for more than two decades. In the Prostate Cancer Prevention Trial, published in NEJM in 2003 Finasteride seemed to prevent or delay the appearance of prostate cancer, but this possible benefit should be weighed against sexual side effects and the increased risk of high-grade prostate cancer. It would therefore be valuable if the current study could include information on Gleason grade to distinguish between indolent and high-grade tumors. 

 The study also lacks information on PSA, prostate volume, and LUTS. The treatment of BPH varies by symptom severity. Alpha-blockers provide rapid symptom relief but do not affect prostate volume. In contrast to alpha-blockers, 5-ARIs have a slow onset of action (three to six months). The effect on symptoms depends on prostate volume, the greater prostate volume, the more and faster effective symptom reduction by 5-ARIs. Men with few symptoms may not receive medical treatment. Accordingly, the three exposure groups are not entirely comparable. Treatment with 5-ARI may reduce prostate volume and thereby PSA level, which could impact detection of prostate cancer in this exposure group. Therefore, it would strengthen the analyses to include information about PSA, prostate volume, and LUTS.

 What is the rationale for presenting univariate estimates for the included covariates?

 Examining various risk factors for prostate cancer was not stated as an aim of the study and it is not useful for selection of potential confounders either as a covariate with a small effect on prostate cancer may still be a confounder if it is prevalent in one of the exposure groups.

Is the work clearly and accurately presented and does it cite the current literature?

Partly

If applicable, is the statistical analysis and its interpretation appropriate?

Yes

Are all the source data underlying the results available to ensure full reproducibility?

No

Is the study design appropriate and is the work technically sound?

Yes

Are the conclusions drawn adequately supported by the results?

Yes

Are sufficient details of methods and analysis provided to allow replication by others?

Yes

Reviewer Expertise:

Clinical Epidemiology

I confirm that I have read this submission and believe that I have an appropriate level of expertise to confirm that it is of an acceptable scientific standard, however I have significant reservations, as outlined above.
